# A phylogenetic and evolutionary analysis of antimycin biosynthesis

**DOI:** 10.1099/mic.0.000572

**Published:** 2017-11-07

**Authors:** Rebecca Joynt, Ryan F. Seipke

**Affiliations:** ^1^​ Faculty of Biological Sciences, University of Leeds, Leeds, LS2 9JT, UK; ^2^​ Astbury Centre for Structural Molecular Biology, University of Leeds, Leeds, LS2 9JT, UK

**Keywords:** *Streptomyces*, secondary metabolism, natural products, evolution of biosynthetic gene clusters, antimycin

## Abstract

*Streptomyces* species and other *Actinobacteria* are ubiquitous in diverse environments worldwide and are the source of, or inspiration for, the majority of antibiotics. The genomic era has enhanced biosynthetic understanding of these valuable chemical entities and has also provided a window into the diversity and distribution of natural product biosynthetic gene clusters. Antimycin is an inhibitor of mitochondrial cytochrome c reductase and more recently was shown to inhibit Bcl-2/Bcl-X_L_-related anti-apoptotic proteins commonly overproduced by cancerous cells. Here we identify 73 putative antimycin biosynthetic gene clusters (BGCs) in publicly available genome sequences of *Actinobacteria* and classify them based on the presence or absence of cluster-situated genes *antP* and *antQ*, which encode a kynureninase and a phosphopantetheinyl transferase (PPTase), respectively. The majority of BGCs possess either both *antP* and *antQ* (L-form) or neither (S-form), while a minority of them lack either *antP* or *antQ* (I_Q_- or I_P_-form, respectively). We also evaluate the biogeographical distribution and phylogenetic relationships of antimycin producers and BGCs. We show that antimycin BGCs occur on five of the seven continents and are frequently isolated from plants and other higher organisms. We also provide evidence for two distinct phylogenetic clades of antimycin producers and gene clusters, which delineate S-form from L- and I-form BGCs. Finally, our findings suggest that the ancestral antimycin producer harboured an L-form gene cluster which was primarily propagated by vertical transmission and subsequently diversified into S-, I_Q_- and I_P_-form biosynthetic pathways.

## Introduction

Microbial natural products, particularly those produced by filamentous *Actinobacteria*, have been a cornerstone of the pharmaceutical industry for more than half a century [1]. The genes encoding natural product biosynthesis are typically grouped together into a gene cluster, which possibly enhances their transmissibility and the evolution of chemical diversity [2]. Little is understood about the forces driving these processes, but access to large datasets of genome sequences provides an opportunity for exploration.

Antimycin-type depsipeptides are a large and diverse family of natural products widely produced by *Streptomyces* species [3]. The family’s namesake, the nine-membered ringed antimycins, were discovered more than 65 years ago [4]. Ring-extended members of this family have also been identified and include: JBIR-06 (12-membered ring), neoantimycin (15-membered ring) and respirantin (18-membered ring) [5–7]. All of these compounds possess antifungal, insecticidal and nematocidal activity, as a result of their ability to inhibit mitochondrial cytochrome c reductase via a conserved 3-formamidosalicylate moiety [8]. Antimycins are used commercially as a fish biocide, but were recently found to be potent and selective inhibitors of the mitochondrial Bcl_2_/Bcl-x_L_-related anti-apoptotic proteins, which are over-produced by cancer cells and confer resistance to apoptotic chemotherapeutic agents [9]. To date, the biosynthesis of antimycins has been reported for a myriad of environmental isolates, but it was not until recently that the hybrid non-ribosomal peptide synthetase (NRPS)/polyketide synthase (PKS) biosynthetic pathway that directs their assembly was revealed in a strain of *Streptomyces albus* [10].

**Fig. 1.**
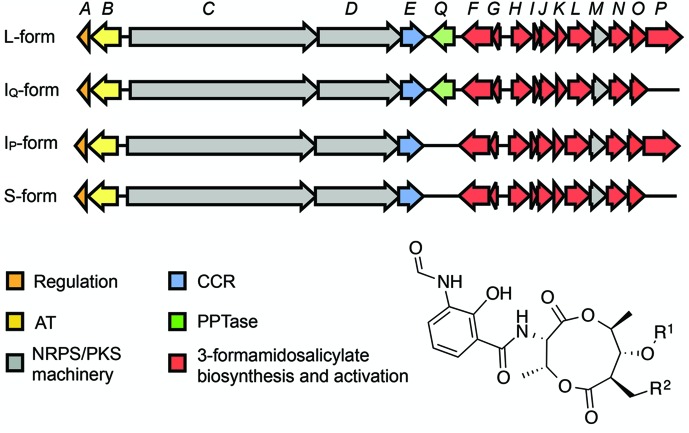
Schematic representation of L-, I_Q_-, I_P_- and S-form antimycin biosynthetic gene clusters. AT, acyltransferase; NRPS, non-ribosomal peptide synthetase; PKS, polyketide synthase; CCR, crotonyl-CoA carboxylase/reductase; PPTase, phosphopantetheinyl transferase. Antimycins: Antimycin A_1_, R^1^=COCH(CH_3_)CH_2_CH_3_, R^2^=(CH_2_)_4_CH_3_; Antimycin A_2_, R^1^=COCH(CH_3_)_2_, R^2^=(CH_2_)_4_CH_3_; Antimycin A_3_, R^1^=COCH_2_CH(CH_3_)_2_, R^2^=(CH_2_)_2_CH_3_; Antimycin A_4_, R^1^=COCH(CH_3_)_2_, R^2^=(CH_2_)_2_CH_3_.

The ~25 kb antimycin biosynthetic gene cluster (BGC) harboured by *S. albus* is composed of 15 genes organized into four polycistronic operons, *antAB*, *antCDE*, *antFG* and *antHIJKLMNO* (Fig. 1) [11]. The biosynthetic gene cluster was recently used as the basis for the reconstitution of antimycin biosynthesis *in vitro* [12, 13] and heterologous production using *Escherichia coli* [14] and *S. coelicolor* [15]. The biosynthesis and activation of the unusual starter unit, 3-formamidosalicylate, is specified by the genes *antFGHIJKLNO* [12, 14, 16]. The di-modular NRPS, AntC and the unimodular PKS, AntD comprise the NRPS-PKS assembly line, while AntE and AntM are crotonyl-CoA carboxylase/reductase and discrete ketoreductase enzymes, respectively, and AntB is an acyltransferase responsible for the acyloxyl moiety and the chemical diversity observed at R^1^ (Fig. 1) [13]. The expression of the antimycin BGC is coordinately regulated with the candicidin BGC by a LuxR-family regulator, FscRI, which activates expression of *antABCDE* [15]. The *antA* gene encodes a cluster-situated extracytoplasmic function RNA polymerase sigma (σ) factor named σ^AntA^, which activates transcription of the *antGF* and *antHIJKLMNO* operons [11].

Intriguingly, subsequent identification of antimycin biosynthetic pathways in other taxa revealed that the BGC possesses up to four architectures [17]: short-form (S-form, 15 genes), intermediate-form (I_Q_- or I_P_-form, 16 genes) and long-form (L-form, 17 genes), based on the absence (S-form) or presence (L-form) of two cluster-situated genes, *antP* and *antQ*, which encode a kynureninase and phosphopantetheinyl transferase, respectively. I-form BGCs harbour either *antP* (I_P_) or *antQ* (I_Q_), but not both (Fig. 1). How the antimycin BGC evolved into these various architectures is an intriguing question and one that we sought to address with this study.

Here we identify 73 antimycin BGCs (five known and 68 putative) in publicly available genome sequences of *Actinobacteria* and evaluate their biogeographical distribution and phylogenetic relationships. Isolation metadata suggest that the antimycin BGC has a large biogeographical range, with isolation of putative antimycin producers from at least five continents. Our phylogenetic analyses support the existence of two distinct clades of antimycin producers and BGCs which delineate S-form from L- and I-form BGCs. Finally, our findings suggest that the ancestral antimycin producer harboured an L-form BGC that was primarily propagated by vertical transmission and subsequently diversified into S-, I_Q_- and I_P_-form biosynthetic pathways.

## Methods

### Identification of putative antimycin biosynthetic gene clusters

The genomes available in GenBank on 9 May 2017 for select genera of *Actinobacteria* (*Actinobactera*, *Actinomadura*, *Actinospica*, *Amycolatopsis*, *Kitasatospora*, *Micromonospora*, *Nocardia*, *Saccharopolyspora*, *Planomonospora*, *Pseudonocardia*, *Salinispora*, *Streptacidiphilus* and *Streptomyces*) were downloaded using the ncbi-genome-download python script provided by Kai Blin available at https://github.com/kblin/ncbi-genome-download. One thousand four hundred and twenty-one genomes were downloaded in total and were subsequently annotated using Prokka 1.12 [18]. Next, annotated GenBank files were modified using the Unix commands grep and sed to move the unique gene prefix generated by Prokka from the ‘/locus_tag’ field to the ‘/gene’ field. A multigeneblast database was created using the makedb programme of multigeneblast version 1.1.13 and the processed GenBank files from above [19]. The genes *antFGHIJKLMNOPQ* of the antimycin BGC from *S. ambofaciens* ATCC 23877 [16] were used as a multigeneblast query with the default settings. The resulting output was inspected manually to identify genomes harbouring a putative antimycin BGC. PROmer [20] and the *S. ambofaciens* antimycin BGC were used to identify contigs comprising antimycin BGCs split across more than one contig. This applied to the following taxa: *S. gancidicus* BKS 13–15, *S.* sp. B9173, *S.* sp. CC71, *S.* sp. HNS054, *S.* sp. IgraMP-1, *S.* sp. MBT28, *S.* sp. NRRL B-24085, *S.* sp. TOR3209 and *S.* sp. SM8. *S. wadayamensis* strain A23, which harbours a putative S-form antimycin BGC, was discarded because it lacked multiple phylosift markers (see below).

### Phylogenetic analyses

In order to infer a species phylogeny, 29 single-copy phylogenetic markers (13 061 nt in total) were identified and extracted using Phylosift version 1.0.1 (Tables S1) [21] and concatenated in the order: DNGNGWU00002, DNGNGWU00003, DNGNGWU00007, DNGNGWU00009, DNGNGWU00010, DNGNGWU00011, DNGNGWU00012, DNGNGWU00014, DNGNGWU00015, DNGNGWU00016, DNGNGWU00017, DNGNGWU00018, DNGNGWU00019, DNGNGWU00021, DNGNGWU00022, DNGNGWU00023, DNGNGWU00024, DNGNGWU00025, DNGNGWU00026, DNGNGWU00027, DNGNGWU00028, DNGNGWU00029, DNGNGWU00030, DNGNGWU00031, DNGNGWU00033, DNGNGWU00034, DNGNGWU00036, DNGNGWU00037, DNGNGWU00040. The concatenated phylogenetic marker sequences were aligned using 16 iterations of muscle [22] and the .fasta format alignment was converted to sequential phylip format using Geneious R8.1.19. Phylogenetic relationships were inferred from this alignment using the web-implementation of PhyML3.0 available at www.atgc-montpellier.fr/phyml/ [23].

In order to infer a phylogeny for putative antimycin BGCs, 10 genes (*antFGHIJKLMNO*) were extracted and aligned using muscle. The resulting alignment was imported into Geneious R8.1.19 and manually trimmed to the same length prior to concatenating sequences in the following gene order: *antFGHIJKLMNO.* The concatenated alignment was then converted to sequential phylip format and a phylogenetic tree was inferred using PhyML3.0 as above.

### Likelihood analysis

Reconstruction of the ancestral state was performed essentially as described previously [24]. Briefly, the trace character function of Mesquite v3.2 [25] was used to infer the ancestral node for the antimycin BGC within the species tree. A categorical character matrix for BGC type was created and likelihood calculations were performed using the Mk1 model.

## Results and discussion

### Identification of putative antimycin biosynthetic gene clusters (BGCs) in *Actinobacteria*

Established and putative antimycin BGCs were previously identified within the genomes of 14 *Streptomyces* species [17, 26]. However, casual analyses of the genome sequences available in GenBank suggested that this number is likely to be far greater. In order to formally assess this possibility, 1421 publically available genome sequences for select *Actinobacteria* genera (i.e. those with a history of natural products production: (*Actinobactera*, *Actinomadura*, *Actinospica*, *Amycolatopsis*, *Kitasatospora*, *Micromonospora*, *Nocardia*, *Saccharopolyspora*, *Planomonospora*, *Pseudonocardia*, *Salinispora*, *Streptacidiphilus* and *Streptomyces*) were downloaded and annotated using Prokka 1.12 [18]. The Prokka annotation enabled the construction of a customized mutligeneblast database, which was subsequently used in conjunction with the *antFGHIJKLMNOPQ* genes from *S. ambofaciens* and multigeneblast 1.1.13 [19] to generate a list of taxa harbouring a putative antimycin BGC. The genes *antFGHIJKLMNO* were selected on the basis that they are essential for antimycin biosynthesis and are conserved in all established antimycin BGCs; *antPQ* were also included in order to permit the tentative classification of gene cluster architecture (see below). Close inspection of gene clusters from the candidate list resulted in the identification of an antimycin BGC in 73 taxa (five known and 68 putative) (Table 1). Among these, five are described as non-*Streptomyces* species: *Saccharopolyspora flava* DSM 44771, *Streptacidiphilus albus* JL83, *Streptacidiphilus albus* NBRC 100918, *Actinospica acidiphila* NRRL B-24431 and *Actinobacteria bacterium* OV320 (Table 1).

**Table 1.** *Actinobacteria* harbouring a putative antimycin biosynthetic gene cluster (BGC)

Organism	Source	Genome accession	Antimycin BGC form*			
			S	I_P_	I_Q_	L
*Actinobacteria bacterium* OV320	*Populus trichocarpa*, Corvallis, Oregon, USA	LJCX00000000.1				
*Actinospica acidiphila*NRRL B-24431	Soil, Gerenzano, Italy	JNYX00000000.1				
*Saccharopolyspora flava* DSM 44771	–	FOZX00000000.1				
*Streptacidiphilus albus* JL83	Soil, Co. Durham, UK	JQML00000000.1				
*Streptacidiphilus albus* NBRC 100918	–	BBPL00000000.1				
*Streptomyces albidoflavus* NRRL B-1271	–	JOII00000000.1				
*Streptomyces albidoflavus* OsiLf-2	Rice, Changsha, Hunan, China	MNPQ00000000.1				
*Streptomyces albidoflavus* R-53649	–	FWFA00000000.1				
*Streptomyces albus* J1074†	–	CP004370.1				
*Streptomyces albus* S4†	*Acromyrmex octospinosus,* Trinidad	CADY00000000.1				
*Streptomyces albus* SM254	Soil, Soudan, Minnesota, USA	CP014485.1				
*Streptomyces ambofaciens* ATCC 23877†	Soil, Picardie, France	CP012382.1				
*Streptomyces ambofaciens* DSM 40697	Soil, Rome, Italy	CP012949.1				
*Streptomyces antibioticus* DSM 41481	Soil, USA	CM007717.1				
*Streptomyces caelestis* NRRL B-24567	–	LGCN00000000.1				
*Streptomyces gancidicus* BKS 13–15	Soil, Odisha, India	AOHP00000000.1				
*Streptomyces griseoflavus* Tu4000	–	ACFA00000000.1				
*Streptomyces griseorubens* strain JSD-1	Soil, Shanghai, China	JJMG00000000.1				
*Streptomyces griseus* subsp. *griseus* NRRL B-2307	–	JNZI00000000.1				
*Streptomyces griseus* subsp. g*riseus* NRRL F-5618	–	JOGU00000000.1				
*Streptomyces griseus* subsp. *griseus* NRRL F-5621	–	JOGN00000000.1				
*Streptomyces griseus* subsp. *griseus* NRRL WC-3066	–	LLZL00000000.1				
*Streptomyces hygroscopicus* subsp. *jinggangensis* 5008	–	CP003275.1				
*Streptomyces hygroscopicus* subsp. *jinggangensis* TL01	–	CP003720.1				
*Streptomyces longwoodensis* DSM 41677	–	LMWS00000000.1				
*Streptomyces* sp. M10	Marine sediment, Dalian, China	AMZL00000000.1				
*Streptomyces mirabilis* OV308	–	JQJV00000000.1				
*Streptomyces pluripotens* MUSC 135	Mangrove, Malaysia	JTDH00000000.1				
*Streptomyces pluripotens* MUSC 137	Mangrove, Malaysia	JUIF00000000.2				
*Streptomyces radiopugnans* CGMCC 4.3519	–	FOET00000000.1				
*Streptomyces sampsonii* KJ40	Poplar tree rhizosphere, Ya’an, Sichuan, China	CP016824.1				
*Streptomyces sp.* 303MFCol5.2	–	ARTR00000000.1				
*Streptomyces* sp. 4F	Soil, Shanghai, China	CP013142.1				
Streptomyces sp. Amel2xC10	–	FWZW00000000.1				
*Streptomyces* sp. AVP053U2	Marine sediment, Avery Point, Connecticut, USA	LMTQ00000000.2				
*Streptomyces* sp. B9173	Ocean sediment, Germany	NAVC00000000.1				
*Streptomyces* sp. BvitLS-983	–	FMCF00000000.1				
*Streptomyces* sp. CC71	Sediment from Churince hydrological system, Cuatro, Cienegas, Coahuila, Mexico	LOSR00000000.1				
*Streptomyces* sp. CdTB01	Soil, Changsha, China	CP013743.1				
*Streptomyces* sp. CNQ431†	Marine sediment, La Jolla, California, USA	JTCK00000000.1				
*Streptomyces* sp. CNY228	–	ARIN00000000.1				
*Streptomyces* sp. F-7	–	FKJH00000000.1				
Streptomyces sp. FR-008	–	CP009802.1				
*Streptomyces* sp. GBA 94–10	*Phakellia ventilabrum*, Trondheimfjord, Norway	CM002271.1				
*Streptomyces* sp. HNS054	Unknown sponge, Fujian, China	LDZX00000000.1				
*Streptomyces* sp. IgraMP-1	–	FMCM00000000.1				
Streptomyces sp. JHA26	Soil, Japan	BDJC00000000.1				
*Streptomyces* sp. KE1	Human skin, New Delhi, India‡	LAYX00000000.1				
*Streptomyces* sp. LaPpAH-202	*Petalomyrmex phylax* plant-ants, Cameroon	ARDM00000000.1				
*Streptomyces* sp. MBT28	Soil, Lanzarote, Canary Islands, Spain	LARV00000000.1				
*Streptomyces* sp. MUSC 125	Mangrove, Malaysia	JUIG00000000.1				
*Streptomyces* sp. NBRC 110035	–	BBNN00000000.1				
*Streptomyces* sp. NBRC 110468	–	BBYG00000000.1				
*Streptomyces* sp. NRRL B-24085	Peanut, South Africa	LJBR00000000.1				
*Streptomyces* sp. NRRL B-2790	–	JOHI00000000.1				
*Streptomyces* sp. NRRL B-3253	–	JOGQ00000000.1				
*Streptomyces* sp. NRRL F-2305	–	JOFW00000000.1				
*Streptomyces* sp. NRRL F-4835	–	JOIEL00000000.1				
*Streptomyces* sp. NRRL F-5008	Farm feed lot waste, Norris Farms, Dickson Mounds, Illinois, USA	JOHW00000000.1				
*Streptomyces* sp. NRRL F-5527	Tobacco, unknown location‡	JOHL00000000.1				
*Streptomyces* sp. NRRL S-1314	–	JOHU00000000.1				
*Streptomyces* sp. NRRL S-37	Soil, El Salvador	JOIZ00000000.1				
*Streptomyces* sp. PVA 94–07	*Phakellia ventilabrum*, Trondheimfjord, Norway	CM002273.1				
*Streptomyces* sp. Root1310	*Arabidopsis thaliana* root, Cologne, Germany	LMEQ00000000.1				
*Streptomyces* sp. Root1319	*Arabidopsis thaliana* root, Cologne, Germany	LMEU00000000.1				
*Streptomyces* sp. Root55	*Arabidopsis thaliana* root, Cologne, Germany	LMFT00000000.1				
*Streptomyces* sp. ScaeMP-6W	–	FMBX00000000.1				
*Streptomyces* sp. SM8†	Sponge, Galway, Ireland	AMPN00000000.1				
*Streptomyces* sp. SolWspMP-5a-2	–	FMCI00000000.1				
*Streptomyces* sp. TOR3209	Tomato rhizosophere, unknown location‡	AGNH00000000.1				
*Streptomyces* sp. URHA0041	Mediterranean grassland soil	JNIH00000000.1				
*Streptomyces* sp. XY152	Soil, Urbana, Illinois, USA	LGDQ00000000.1				
*Streptomyces yokosukanensis* DSM 40224	Soil, Yokosuka City, Japan	LMWN00000000.1				

*See Fig. 1 and manuscript text for definitions of S-, I_P_-, I_Q_- and L-form BGCs.

†Antimycin production verified experimentally.

**Fig. 2.**
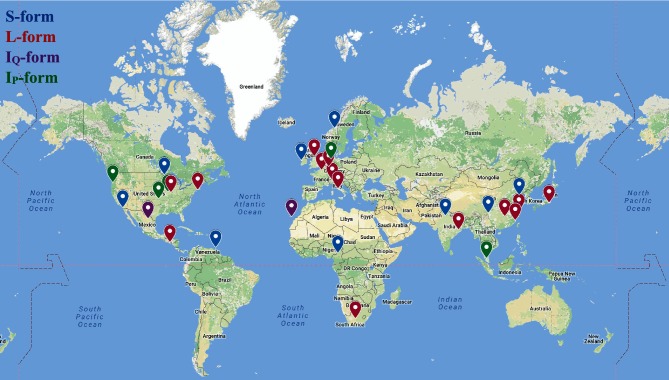
Geographical distribution of 38 *Actinobacteria* harbouring a putative antimycin biosynthetic gene cluster. Map pins are colour-coded based on gene cluster classification. The map is available here: https://drive.google.com/open?id=1rXUFJXSt8szUYMuCJExQbEP4X1k.

‡Location not mapped in Fig. 2.

Inspection of loci identified as encoding 3-formamidosalicylate biosynthetic genes revealed a few noteworthy peculiarities. *Streptomyces albus* subsp. *albus* strain NRRL B-2513 possesses a clear 3-formamidosalicylate locus, but lacks *antM* and the core NRPS-PKS biosynthetic machinery at this locus or elsewhere in the genome; and *Streptomyces phaeoluteigriseus* strain DSM 41896 has endured at least two frameshift mutations in *antD,* which presumably render it non-functional. In addition, *Streptomyces lincolnensis* strain NRRL 2936, *Streptomyces* sp. yr375 and *Streptomyces* sp. ERV7 each harbour the same antimycin-like BGC. However, gene rearrangement and insertion is evident: for example, a small locus of fatty acid anabolism genes has been inserted between the 3-formamidosalicylate biosynthetic genes and the NRPS-PKS machinery, suggesting that the biosynthetic pathway may not in fact produce antimycins. These strains and BGCs were discarded as a consequence of these peculiarities.

### Classification of antimycin BGCs

Antimycin BGCs exist in four architectures, and the gene clusters identified here were classified as short-form (S-form, 15 genes), intermediate-form (I_P_- or I_Q_-form, 16 genes) and long-form (L-form, 17 genes), based on the absence (S-form) or presence (L-form) of two cluster-situated genes: *antP,* which encodes a kynureninase (InterPro ID, IPR010111) involved in the production of the 3-formamidosalicylate starter unit, and *antQ,* which is a phosphopantetheinyl transferase (InterPro ID, IPR0082788) responsible for the post-translational modification of the NRPS/PKS assembly line to its pantetheinylated form [17]. The organization of the genes within antimycin BGCs shows 100 % synteny (Fig. 1), and their functions are described in Table 2. Annotation of the putative biosynthetic pathways identified above resulted in the classification of 25 S-form, 13 I_P_-form, five I_Q_-form and 30 L-form antimycin BGCs (Table 1).

**Table 2.** Functions of proteins encoded by antimycin BGCs

Protein	Function
AntA	Extracytoplasmic function RNA polymerase sigma factor
AntB	Acyltransferase
AntC	Dimodular non-ribosomal peptide synthetase
AntD	Unimodular polyketide synthase
AntE	Crotonyl-CoA reductase/decarboxylase
AntF	Acyl-CoA ligase
AntG	Peptidyl carrier protein
AntH	Multi-component oxygenase
AntI	Multi-component oxygenase
AntJ	Multi-component oxygenase
AntK	Multi-component oxygenase
AntL	Multi-component oxygenase
AntM	Ketoreductase
AntN	Tryptophan 2,3-dioxygenase
AntO	*N*-formylase
AntP	Kynureninase
AntQ	Phosphopantetheinyl transferase

The first step in the biosynthesis of the 3-formamidosalicylate starter unit is oxygenation of the indole ring of tryptophan by the AntN tryptophan 2,3-dioxygenase, resulting in kynurenine. This is then presumably converted to anthranilate by the AntP kynureninase (harboured by L- and I_P_-forms), whereas in the S- and I_Q_-forms this functionality is provided by the housekeeping kynureninase involved in normal tryptophan catabolism [17]. AntF then activates anthranilate, which is subsequently loaded onto the AntG carrier protein. An important point is that although the L- and I_P_-forms possess AntP and thus have a ‘dedicated’ source of anthranilate, all variants of the antimycin biosynthetic pathway are able to access anthranilate from the ‘core’ anthranilate pool within the cell, which is corroborated by feeding studies with exogenous fluoroanthranilates [16]. The maintenance of *antP* by L- and I_P_-forms and its loss by S- and I_Q_-forms may be driven by physiological differences, for instance the availability of cytosolic anthranilate. It is perhaps not surprising that AntQ is not essential in the S- and I_P_-forms; in fact, most NRPS and PKS biosynthetic systems lack a cluster-situated PPTase and are dependent on the promiscuity of one or more PPTase enzymes encoded elsewhere in the genome. This is clearly the case for S- and I_P_-form antimycin BGCs, but it may not be so for L-form antimycin BGCs, as antimycin production was abolished in an *S. ambofaciens ∆antQ* mutant [27]. The contextual requirement of *antP* and *antQ* for antimycin biosynthesis creates the opportunity for divergent evolution of the antimycin BGC.

### Biogeographical distribution of antimycin biosynthesis

The biogeography of natural products biosynthesis is an emerging area and one that can not only guide future natural products bioprospecting campaigns, but which enables formulation of interesting questions in chemical microbial ecology [24, 28]. Thus, we curated isolation metadata for putative antimycin producers to ascertain any patterns in source material or its geographical origin. The breadth of data varied considerably, but a source and/or country location was available in GenBank or within the literature for 38 out of 73 strains (Table 1). Sample collection data were plotted onto a world map and pins were colour-coded based on gene cluster architecture. Inspection of the resulting map did not show an obvious link between gene cluster architecture and geographical location, but did reveal that putative antimycin producers have been isolated from a relatively large geographical area, including at least five of the seven continents: Africa, Asia, Europe, North America and South America (Fig. 2). Only a single strain originates from the southern hemisphere, which is surprising; however, this is likely a consequence of the inherent limitations of the dataset. Like geographical location, gene cluster architecture and isolation source material do not appear to be related, however, but as anticipated, many strains originate from various soil ecosystems (17 in total) or marine sediments (four in total), which supports the long-standing view that these niches are rich sources of bioactive metabolites. Interestingly, 18 of the strains were isolated from plants, sponges or insects, suggesting that they may be involved in symbioses, which is in line with the increasing number of reports implicating antibiotic-producing strains as defensive symbionts of higher organisms [29, 30]. Overall, these data suggest that antimycin-producing *Actinobacteria* are likely distributed worldwide, which may reflect the significance of producing an inhibitor of eukaryotic cytochrome c reductase in diverse niches.

### Distribution of antimycin BGCs within *Actinobacteria*

**Fig. 3.**
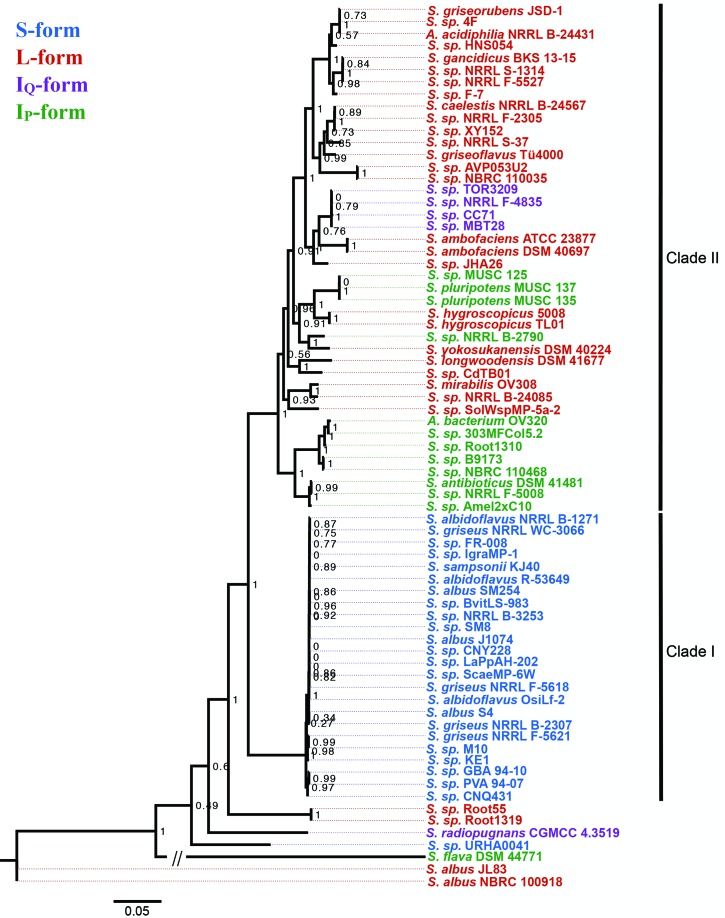
Maximum likelihood phylogeny of 73 *Actinobacteria* analysed in this study. The phylogeny is based on 29 concatenated ribosomal protein DNA sequences. SH-like support values are indicated at nodes as decimal values. Colours indicate gene cluster classification. The scale bar represents 5 % sequence divergence.

The collection of putative antimycin BGCs identified here provides an opportunity to further explore their phylogenetic and evolutionary relationships. A multi-locus phylogeny was reconstructed in order to evaluate the taxonomic distribution of antimycin BGCs. Phylosift was used to identify and extract phylogenetic markers from the genome of each microbe described in Table 1. This resulted in the identification of 29 phylogenetic markers present in single copy in each taxon (see Table S1, available in the online version of this article for description of markers). The markers were concatenated, aligned (length 13 119 nt) and used to infer a maximum likelihood (ML) phylogenetic tree (Fig. 3). Inspection of the resulting phylogeny suggested that six strains have been taxonomically mis-assigned. For instance, *Actinospica acidiphila* strain NRRL B-24431 and *Actinobacteria bacterium* strain OV320 group closely with *Streptomyces* species placed within the interior of the tree, and are therefore likely to be members of the genus *Streptomyces* (Fig. 3). Additionally, four taxa designated as *S. griseus* (*S. griseus* subsp. *griseus* NRRL B-2307, *S. griseus* subsp. g*riseus* NRRL F-5618, *S. griseus* subsp. *griseus* NRRL F-5621, *S. griseus* subsp. *griseus* NRRL WC-3066) group within the *S. albus* J1074 clade and are thus likely strains of this species and not strains of the streptomycin producer, *S. griseus*.

Next, the phylogenetic tree was colour-coded based on the gene cluster architectures determined above. The sixth bifurcation divides the tree into two major clades, one of which contains 24 of the 25 strains harbouring an S-form antimycin BGC (Clade I), and a second that contains strains harbouring exclusively I_P_-, I_Q_- and L- form antimycin BGCs (Clade II, Fig. 3). Within Clade I, all 24 S-form strains are closely related to *S. albus* J1074. Within Clade II, 50 % of the L-form antimycin BGCs are harboured by taxa that comprise a single subclade near the top of the tree that includes several isolates from the United States Department of Agriculture NRRL collection, as well as *A. acidiphila* NRRL B-24431 (Fig. 3). The remaining L-form antimycin BGCs are harboured by small groupings of strains as well as singletons, and interspersed amongst L-form taxa are those that harbour I_Q_- and I_P_-form gene clusters (Fig. 3). Interestingly, seven strains fall outside of Clades I and II: *S.* sp. URHA0041 (S-form), *S. radiopugnans* CGMCC 4.3519 (I_Q_-form), *S. flava* DSM 44771 (I_P_-form), and *S.* sp. Root1319, *S.* sp. Root55, *S. albus* JL83 and *S. albus* NRBC 100918 (all L-form), which suggests that either these strains may be closely related to the ancestral antimycin producer or the genes for antimycin biosynthesis were horizontally acquired by these strains (Fig. 3).

### Antimycin BGC phylogeny

**Fig. 4.**
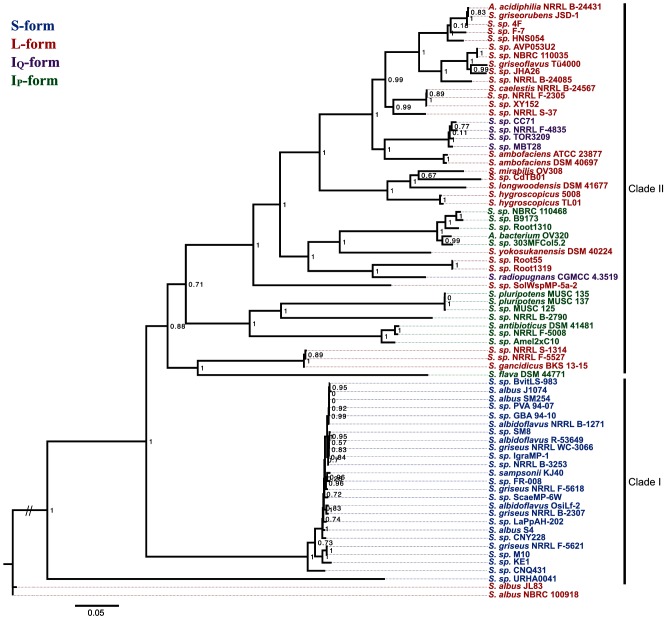
Maximum likelihood phylogeny of the 73 antimycin biosynthetic gene clusters analysed in this study. The phylogeny is based on concatenated *antFGHIJKLMNO* DNA sequences. SH-like support values are indicated at nodes as decimal values. Colours indicate gene cluster classification. The scale bar represents 5 % sequence divergence.

A ML phylogeny was inferred from concatenated sequences of *antFGHIJKLMNO* (alignment length 9736 nt) and colour-coded based on gene cluster architecture as above, in order to evaluate the evolutionary relationships of antimycin BGCs. These genes were selected because they are conserved in all BGCs and changes to their DNA sequence should not impact antimycin biochemical diversity. The third bifurcation divides the tree into two major clades, Clade I which harbours only S-form antimycin BGCs and Clade II which harbours exclusively L-, I_Q_- and I_P_-form antimycin BGCs (Fig. 4). As with the phylosift phylogeny above, 24 of the 25 S-form antimycin BGCs comprise a closely related clade, which was anticipated after the revelation that all of these BGCs are harboured by strains closely related to *S. albus* J1074 (Fig. 4). L-form antimycin BGCs appear throughout Clade II; 14 of the 30 L-forms clade together at the top of the tree and the majority of the remainder comprise smaller groupings consisting of five, three and two members with two singletons. Four of the five I_Q_-form BGCs clade together and are flanked on either side by L-form antimycin BGCs. I_P_-form antimycin BGCs form two clades in the centre of the tree comprising a total of 12 of the 13 gene clusters. Overall, the tree highlights that phylogenetic placement of antimycin BGCs is linked to their gene cluster architecture in the majority of cases. There are three notable exceptions to this – *S. albus* JL83, *S. albus* NBRC 100918 and *S.* sp. URHA0041 do not group into either Clade I or II and their basal position within the phylogeny may suggest a close relationship with the ancestral antimycin BGC (Fig. 4).

### Antimycin BGC evolution

**Fig. 5.**
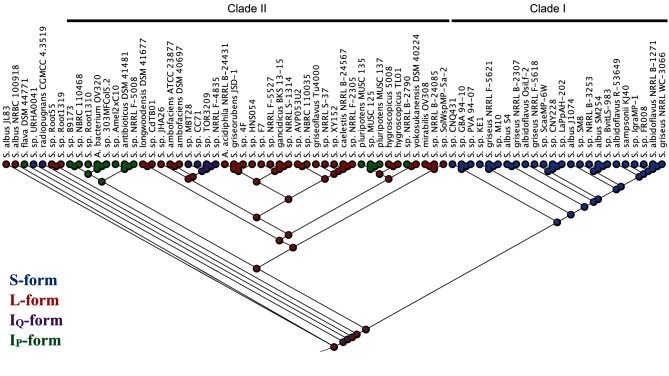
Likelihood analysis of ancestral antimycin BGC architectures. Filled circles are colour-coded to represent the proportional likelihood of BGC architecture at ancestral nodes within the species phylogeny depicted based on the ribosomal protein DNA sequences in Fig. 3.

There are obvious similarities between the species and BGC trees. For instance, both trees bifurcate to separate S-form from I_Q_-, I_P_- and L-form taxa and BGCs. This, combined with the presence of gene cluster architecture subclades in both trees, suggests that speciation has been the primary driver for dissemination of the antimycin BGC. With respect to the both the species and BGC trees, it is reasonable to propose that antimycin biosynthesis evolved once and that the ancestral antimycin producer harboured an L-form BGC. To test this hypothesis, a likelihood analysis was used to predict the ancestral node for each architecture of the antimycin BGC based on its distribution within the phylosift phylogeny. As expected, the likelihood analysis predicted that the ancestral antimycin producer harboured an L-form BGC (Fig. 5). This supports a model in which loss of *antP* and/or *antQ*, rather than their frequent independent acquisition, resulted in diversification of gene cluster architecture. The ancestral antimycin producer likely gave rise to *S. albus* JL83, *S. albus* NBRC 100918 and *S.* sp. URHA0041, but *S. sp.* URHA0041 lost *antP* and *antQ* after speciation. The same ancestral L-form strain presumably also seeded Clade I where *antP* and *antQ* were lost in the process, but were retained during the genesis of Clade II. One major diversification event likely occurred to give rise to most of the I_P_-form antimycin BGCs, but a second diversification event appears to have occurred where *antQ* was lost by the ancestor of *S.* sp. NRRL B-2790, *S. pluripotens* MUSC 135, *S. pluripotens* MUSC137 and *S.* sp. MUSC125, but was retained by the two *S. hygroscopicus* strains with which the aforementioned clade. Finally, four of the five I_Q_-form BGCs are derived from the L-form ancestor of *S. ambofaciens* ATCC 23877 and *S. ambofaciens* DSM 40697.

Although the phylosift and BGC trees are consistent with that proposed above, there is noticeable discordance between the two phylogenies. The most profound of these are highlighted below. Three closely related strains, *S. gancidicus*, *S.* sp. NRRL S-1314 and *S*. sp. NRRL F-5527, group within the large subclade of L-form BGCs in Clade II of the phylosift tree, but shift to occupy a distantly related lineage in Clade II of the *antFGHIJKLMNO* phylogeny. This suggests that the parent of the *S. gancidicus* subclade likely received its L-form gene cluster by horizontal gene transfer. The same is also likely true for *S.* sp. NRRL B-24085, which is located within the centre of Clade II in the phylosift tree, but then joins the large L-form subclade of Clade II in the BGC tree. Interestingly, *Sacharropolyspora flava* DSM 44771, which is an ‘outlier’ in the phylosift tree (i.e. does not group within Clade I or II), becomes part of Clade II in the BGC tree and shares an ancestral node with the three-membered *S. gancidicus* subclade described above. This suggests that *S. flava* and the *S. gancidicus* subclade likely received their antimycin BGC from the same or a closely related ancestor, and is consistent with the hypothesis that *S. flava* originally harboured an l-form BGC, but independently lost *antQ* to give rise to its I_P_-form BGC. In addition, three other outliers from the phylosift tree group within Clade II of the BGC tree: *S.* sp. Root1319, *S.* sp. Root55 and *S. radiopugnans*, which suggests that their antimycin BGC was horizontally acquired and moreover that *S. radiopugnans* probably independently evolved an I_Q_-form antimycin BGC from the clade founder.

### Conclusions and perspectives

**Fig. 6.**
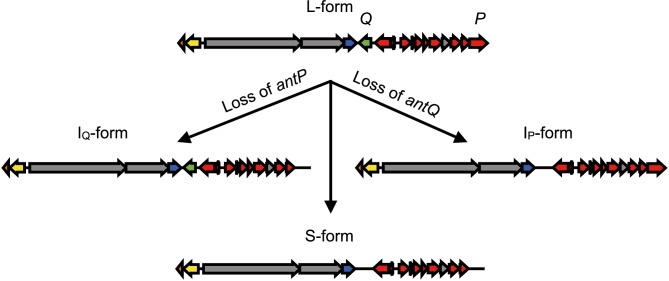
Proposed evolutionary path of the antimycin BGC. The ancestral antimycin producer likely harboured an l-form gene cluster which independently lost either *antP*, *antQ* or both of these genes to give rise to the I_Q_-, I_P_- and S-form gene clusters, respectively.

In this study, 73 antimycin BGCs were identified in the genome sequences of *Actinobacteria* deposited in GenBank. The isolation data for these strains indicate that antimycin-producing actinomycetes are likely globally distributed, highlighting a potentially important role for inhibiting cytochrome c reductase in diverse ecological niches. The majority of the antimycin BGCs identified contained both the *antP* kynureninase and the *antQ* PPTase (L-form), or neither of these (S-form), while a minority of the gene clusters lacked either the *antP* or *antQ* (I_Q_- or I_P_-form, respectively). Phylogenetic analyses revealed two distinct lineages separating S-form from L-, I_Q_- and I_P_-form strains and BGCs, and although a handful of taxa appear to have acquired the antimycin BGC via horizontal gene transfer, the primary means for dissemination of the gene cluster is vertical transmission. The contextual requirement of *antP* and *antQ* presumably permitted divergent evolution of the antimycin biosynthetic pathway. We propose that the ancestral antimycin producer harboured an L-form antimycin BGC, which spawned two main clades, one composed of S-forms that lack both *antP* and *antQ*, and one composed of L-forms with distinct subclades of I_P_- and I_Q_-forms (Fig. 6).

## Supplementary Data

16 Supplementary File 1
