# Supplementary material for: A phylogenetic and evolutionary analysis of antimycin biosynthesis
Source: Microbiology (Reading). 2017 Nov 7;164(1):28–39. doi: 10.1099/mic.0.000572 (PMC5883857; doi:10.1099/mic.0.000572)
Supplement: Supplementary File 1 [file mic-164-28-s001.pdf]

Table S1. Phylogenetic markers identified by Phylosift 1.0.1 and used in this study

Phylosift marker*	Description
DNGNGWU00002	Ribosomal protein S10
DNGNGWU00003	Ribosomal protein L1
DNGNGWU00007	Ribosomal protein L22
DNGNGWU00009	Ribosomal protein L4/L1e
DNGNGWU00010	Ribosomal protein L2
DNGNGWU00011	Ribosomal protein S9
DNGNGWU00012	Ribosomal protein L3
DNGNGWU00014	Ribosomal protein L14b/L23e
DNGNGWU00015	Ribosomal protein S5
DNGNGWU00016	Ribosomal protein S19
DNGNGWU00017	Ribosomal protein S7
DNGNGWU00018	Ribosomal protein L16/L10E
DNGNGWU00019	Ribosomal protein S13
DNGNGWU00021	Ribosomal protein L15
DNGNGWU00022	Ribosomal protein L25/L23
DNGNGWU00023	Ribosomal protein L6
DNGNGWU00024	Ribosomal protein L11
DNGNGWU00025	Ribosomal protein L5
DNGNGWU00026	Ribosomal protein S12/S23
DNGNGWU00027	Ribosomal protein L29
DNGNGWU00028	Ribosomal protein S3
DNGNGWU00029	Ribosomal protein S11
DNGNGWU00030	Ribosomal protein L10
DNGNGWU00031	Ribosomal protein S8
DNGNGWU00033	Ribosomal protein L18P/L5E
DNGNGWU00034	Ribosomal protein S15P/S13e
DNGNGWU00036	Ribosomal protein S17
DNGNGWU00037	Ribosomal protein L13
DNGNGWU00040	Ribosomal protein L24

*More detail about Phylosift markers is available at
https://phylosift.wordpress.com/tutorials/scripts-markers/
